# Supplementary figures and images for: Spatially Structured Optical Pump for Laser Generation Tuning
Source: Nanomaterials (Basel). 2023 Dec 23;14(1):49. doi: 10.3390/nano14010049 (PMC10780650; doi:10.3390/nano14010049)

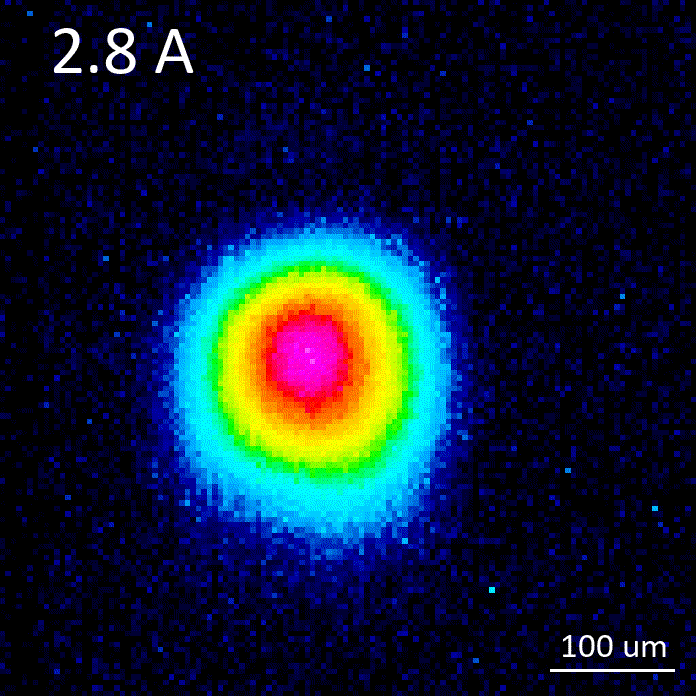

Supplement: Supplementary file 1 [file nanomaterials-14-00049-s001.zip › nanomaterials-2713897-supplementary.gif]
